# Serum Proteomic Profiling to Identify Biomarkers of Premature Carotid Atherosclerosis

**DOI:** 10.1038/s41598-018-27265-9

**Published:** 2018-06-15

**Authors:** Santosh D. Bhosale, Robert Moulder, Mikko S. Venäläinen, Juhani S. Koskinen, Niina Pitkänen, Markus T. Juonala, Mika A. P. Kähönen, Terho J. Lehtimäki, Jorma S. A. Viikari, Laura L. Elo, David R. Goodlett, Riitta Lahesmaa, Olli T. Raitakari

**Affiliations:** 1 Turku Centre for Biotechnology, University of Turku and Åbo Akademi University, Turku, Finland; 2 Department of Medicine, University of Turku and Division of Medicine, Turku University Hospital, Turku, Finland; 3 Department of Clinical Physiology, Tampere University Hospital and University of Tampere, Faculty of Medicine, Tampere, Finland; 4 Department of Clinical Chemistry, Fimlab Laboratories and Finnish Cardiovascular Research Center-Tampere, Faculty of Medicine and Life Sciences, University of Tampere, Tampere, Finland; 5 Department of Pharmaceutical Science, University of Maryland, Baltimore, Maryland USA; 6 Research Centre of Applied and Preventive Cardiovascular Medicine, University of Turku, Turku, Finland; 7 Department of Clinical Physiology and Nuclear Medicine, Turku University Hospital, Turku, Finland

## Abstract

To evaluate the presence of serum protein biomarkers associated with the early phases of formation of carotid atherosclerotic plaques, label-free quantitative proteomics analyses were made for serum samples collected as part of The Cardiovascular Risk in Young Finns Study. Samples from subjects who had an asymptomatic carotid artery plaque detected by ultrasound examination (N = 43, Age = 30–45 years) were compared with plaque free controls (N = 43) (matched for age, sex, body weight and systolic blood pressure). Seven proteins (p < 0.05) that have been previously linked with atherosclerotic phenotypes were differentially abundant. Fibulin 1 proteoform C (FBLN1C), Beta-ala-his-dipeptidase (CNDP1), Cadherin-13 (CDH13), Gelsolin (GSN) and 72 kDa type IV collagenase (MMP2) were less abundant in cases, whereas Apolipoproteins C-III (APOC3) and apolipoprotein E (APOE) were more abundant. Using machine learning analysis, a biomarker panel of FBLN1C, APOE and CDH13 was identified, which classified cases from controls with an area under receiver-operating characteristic curve (AUROC) value of 0.79. Furthermore, using selected reaction monitoring mass spectrometry (SRM-MS) the decreased abundance of FBLN1C was verified. In relation to previous associations of FBLN1C with atherosclerotic lesions, the observation could reflect its involvement in the initiation of the plaque formation, or represent a particular risk phenotype.

## Introduction

Atherosclerotic cardiovascular diseases are amongst the leading causes of death globally^1^. Atherosclerosis is characterized by the accumulation of pro-atherogenic lipoprotein particles in the sub-endothelial space of large- and medium-sized arteries^2^. The process is initiated by the trapping of apolipoprotein B particles in arterial intima by proteoglycans, followed by subsequent modifications, such as aggregation and oxidation, leading to the development of atherosclerotic plaques^3^. The rupture of a critically located atherosclerotic plaques can result in myocardial infarction and stroke^4^.

Ultrasound examination can be used to identify thickening of the intima-media in the carotid arteries and is used to detect and monitor both subclinical and clinical atherosclerosis^5^. Although it is unclear how the diffuse thickening of carotid artery wall represents the subclinical phase, there is a consensus that local carotid plaques, defined as distinct protrusions from the carotid vessel wall into the lumen, are indicative of specific phenotypic changes associated with an active atherosclerotic process^6^. As there is compelling evidence that the atherosclerotic process often starts early in life and may remain asymptomatic for several decades^7^, the study of young and middle-aged adults with non-obstructive carotid plaques provides an opportunity to explore biomarkers for early-stage preclinical atherosclerosis. Such markers may have potential in diagnostics and in understanding the disease etiology. Since atherosclerosis is a circulatory manifestation, the extracellular proteome, which includes proteins like collagens, elastin, proteoglycans, lipoproteins and glycoproteins, is an important analytical target^8,9^. Accordingly, a number of studies have used serum and plasma to establish or identify protein markers for atherosclerosis^10,11^. These have ranged from targeted comparisons, i.e. based on prior hypothesis, through to untargeted profiling discovery measurements^12,13^. As an example of the former, Malaud *et al*. determined proteomic profiles of atherosclerotic lesions, followed by Luminex immunoassays of likely targets in blood from the same subjects^14^. Using a discovery approach, DeGraba *et al*. employed surface-enhanced laser desorption/ionization (SELDI)-time of flight (TOF) mass spectrometry to identify distinguishing proteomics patterns from serum samples of atherosclerotic and non-atherosclerotic groups^12^. Employing a multi-faceted discovery strategy, Kristensen *et al*. compared subjects with different circulatory diseases using a combination of immuno-affinity depletion, isobaric labeling, and an additional consideration of phosphorylated and sialyated peptides. Vinculin was identified as a novel marker of acute coronary syndrome^13^.

In contrast to the published studies, in which the comparisons have mostly addressed advanced atherosclerotic phenotypes, we have analyzed serum samples taken early on from the subjects with a non-obstructive plaque in their carotid artery along with the matched controls. The samples were collected as part of The Cardiovascular Risk in Young Finns Study (YFS), which was established to investigate how childhood lifestyle, biological and psychological measures contribute to cardiovascular risk. Follow-up data on cardiovascular risk factors (e.g. body weight, blood pressure and other biochemical parameters) have been periodically determined at three to six years intervals from over two thousand YFS participants during the past thirty years. Ultrasound assessment of carotid plaque formation has also been performed during the last fifteen years^15^. On the basis of these ultrasound measurements, serum samples were selected from subjects (N = 43), in whom early signs of plaque development was discerned, together with the equivalent material from carefully matched controls (N = 43). With the view to identify markers of disease risk and onset, we have applied a label-free quantitative mass spectrometry approach to analyze this unique sample set^16^. Selected reaction monitoring mass spectrometry (SRM-MS) was subsequently used for the verification of the observed differences. The label-free strategy employed was advantageous in terms of ease of implementation and scalability. Additionally, targeted mass spectrometry-based validation assays could be quickly developed from the discovery data and applied to validate the results.

## Results

### Discovery phase of premature carotid atherosclerosis biomarkers

**Figure 1:**
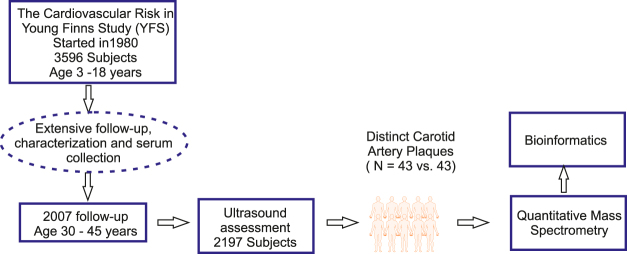
A Schematic representation of the study. Serum samples were selected from subjects of the YFS prospective study cohort on the basis of ultrasonic assessment of carotid artery intima-media thickness, with control samples selected on the basis of age, gender, body weight and systolic blood pressure (N = 43 vs. 43). Mass spectrometry-based serum proteomics analysis of depleted serum was used. The representation of human outline was adapted from Motifolio (www.motifolio.com).

Label-free quantitative proteomics was performed on serum samples obtained from 43 subjects who developed premature carotid artery plaques and 43 matched controls (Figure 1 and Table 1). Overall 296 proteins were detected with more than 1 peptide, according to the defined filtering criteria (see Methods section). For statistical analysis, 249 proteins with valid values in at least 50% of the samples were further considered (Supplementary Table 1). Notably, whilst atherosclerosis is characterized as an inflammatory disease, our clinical data from this study of the early phases of plaque formation revealed that there were no differences in the level of inflammatory C-reactive protein between cases and controls (Table 1).

**Table 1:** Clinical parameters at the time of plaque detection.

Variables	Control			Case		
	Average	SD	N	Average	SD	N
IMT	0.55	0.04	43	0.7	0.1	43
BMI, kg/m^2^	25.72	3.84	43	25.69	3.33	43
Systolic BP, mm Hg	124.02	11.7	43	126.45	13.58	43
Diastolic BP, mm Hg	76.29	10.14	43	79.2	12.31	43
Triglycerides, mmol/L	1.33	0.65	43	1.61	1	43
APOA1	1.65	0.24	43	1.55	0.23	43
APOB	1.04	0.25	43	1.2	0.26	43
CRP, mg/L	1.73	2.19	43	1.66	3.25	43
HDL-cholesterol, mmol/L	1.39	0.29	43	1.23	0.34	43

IMT, indicates intima-media thickness; BMI, body mass index; BP, blood pressure; APOA1, apolipoprotein A1; APOB, apolipoprotein B100; CRP, C-reactive protein; HDL, high-density lipoprotein; N, number of participants.

Note: From the cases and controls there were 7 and 5 smokers, respectively (including two case-control pairs).

**Figure 2:**
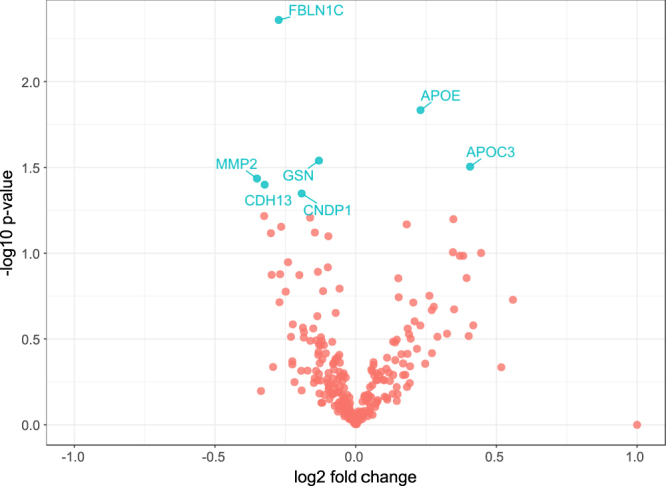
A volcano plot showing the differential level of proteins between subjects who developed plaques and controls (N = 43 vs. 43). Each point represents a protein. The FDR corrected P-values were calculated by permutation (1000 times). Only the difference in FBLN1C was significant after multiple hypothesis testing, whilst the other labeled proteins had p < 0.05.

The comparison of the samples from the plaque bearing subjects and their controls revealed the differential abundance of seven proteins (p < 0.05) as shown in Table 2 and depicted as a volcano plot in Fig. 2. Fibulin 1 proteoform C (FBLN1C), Beta-ala-his-dipeptidase (CNDP1), Cadherin-13 (CDH13), Gelsolin (GSN) and 72 kDa type IV collagenase (MMP2) were lower in abundance in cases, whilst apolipoprotein C-III (APOC3) and apolipoprotein E (APOE) were more abundant. After correction for multiple hypothesis testing only the difference in the FBLN1C levels was statistically significant (FDR < 0.05). On the basis of the known genetic associations, we evaluated the APOE genotype data but found no difference in the frequency of the carotid atherosclerosis risk related alleles between the case and control groups (Supplementary Fig. S2 and Supplementary Table 2).

**Table 2:** List of proteins found to be significantly differentially abundant between cases and their matched controls.

UniProt IDs	Protein name	No. of unique + razor peptides	Sequence coverage (%)	Log 2 fold change	p value	FDR
P23142-4	Fibulin-1 proteoform C	6	36.6	−0.27	0.004	0
P02649	Apolipoprotein E	34	80.1	0.22	0.01	NS
P06396	Gelsolin	70	75.8	−0.13	0.03	NS
P02656	Apolipoprotein C-III	8	62.6	0.4	0.03	NS
P08253	72 kDa type IV collagenase	3	7.4	−0.35	0.04	NS
P55290	Cadherin-13	5	9.3	−0.32	0.04	NS
Q96KN2	Beta-Ala-His dipeptidase	29	57.6	−0.19	0.04	NS

FDR = 0 represents a value < 0.0001, NS = not significant.

### Machine learning classification

**Figure 3:**
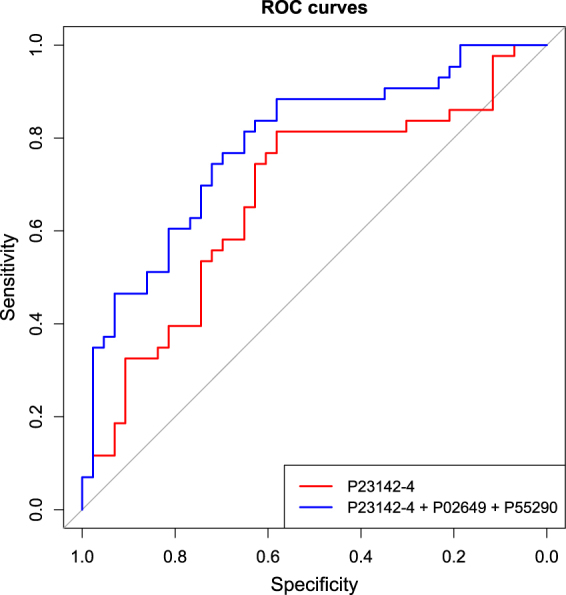
Receiver operating characteristics (ROC) curve for the determined panel of three proteins. P23142-4 (FBLN1C) alone classified cases from controls with AUROC = 0.67 (95% CI: 0.56–0.79). The addition of P02649 (APOE) and P55290 (CDH13) to FBLN1C significantly improved AUROC to 0.79 (95% CI: 0.69–0.88) (p = 0.03). (N = 43 vs. 43).

To gain an overview of whether there was a panel of proteins that could be used to distinguish the subjects we applied Lasso penalized logistic regression to the serum proteomics data. On the basis of this, a panel of three proteins, FBLN1C, APOE and CDH13, was observed to provide the best discrimination between the cases and controls. With the inclusion of APOE and CDH13, there was a statistically significant improvement in the AUROC (0.79, 95% CI: 0.69–0.88, p = 0.03) (Fig. 3). FBLN1C alone classified cases from controls with an AUROC value of 0.67 (95% CI: 0.56–0.79).

### SRM Verification

**Figure 4:**
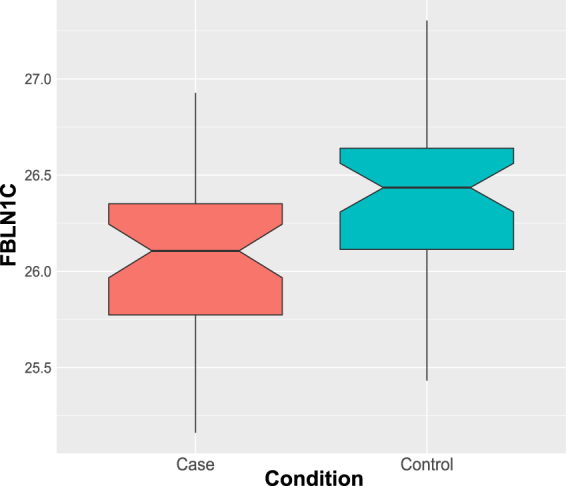
Box-Whisker plot showing relative abundance of FBLN1C measured using SRM-MS assay in the YFS cohort (control, N = 43; cases, N = 43). The relative abundance of FBLN1C was found to be lower in cases than controls (Adjusted p-value = 0.039). Mass spectrometry-based serum proteomics analysis of undepleted serum was used.

SRM measurements were performed for the seven proteins that were indicated to be differentially abundant in the initial profiling data. Additional targeted measurements were made for two housekeeping proteins (selected based on their consistency in our data), APOB (an established CVD risk factor) and standard retention time peptides (iRT)^17^ (Table 3). The analysis supported the downregulation of FBLN1C by a ratio of 0.85 (99% CI: 0.73–0.98; FDR < 0.05) in cases compared to their matched controls (Fig. 4). There were, however, no significant differences observed in any of the other targets. The SRM measurements for the panel did not improve the classification of the cases from controls as in the discovery phase (Supplementary Figure S3).

**Table 3:** List of proteins along with their proteotypic peptides used for the SRM-MS assay.

Uniprot IDs	Protein name	Peptide Sequence	Peptide Sequence Length
P04114	Apolipoprotein B-100	EYSGTIASEANTYLNSK	17
P04114	Apolipoprotein B-100	ENFAGEATLQR	11
P04114	Apolipoprotein B-100	EVGTVLSQVYSK	12
P02656	Apolipoprotein C-III	DALSSVQESQVAQQAR	16
P02656	Apolipoprotein C-III	GWVTDGFSSLK	11
P04217	Alpha-1B-glycoprotein	NGVAQEPVHLDSPAIK	16
P04217	Alpha-1B-glycoprotein	SGLSTGWTQLSK	12
P09871	Complement C1s subcomponent	IIGGSDADIK	10
P09871	Complement C1s subcomponent	TNFDNDIALVR	11
P09871	Complement C1s subcomponent	GDSGGAFAVQDPNDK	15
P23142-4	Fibulin-1 proteoform C	DLLLTVK	7
P23142-4	Fibulin-1 proteoform C	HGTVSSFVAK	10
P02649	Apolipoprotein E	LGPLVEQGR	9
P02649	Apolipoprotein E	LEEQAQQIR	9
P06396	Gelsolin	AGALNSNDAFVLK	13
P06396	Gelsolin	TPSAAYLWVGTGASEAEK	18
P08253	72 kDa type IV collagenase	IIGYTPDLDPETVDDAFAR	19
P08253	73 kDa type IV collagenase	AFQVWSDVTPLR	12
P55290	Cadherin-13	DVGKVVDSDRPER	13
P55290	Cadherin-13	INENTGSVSVTR	12
Q96KN2	Beta-Ala-His dipeptidase	EWVAIESDSVQPVPR	15
Q96KN2	Beta-Ala-His dipeptidase	FRQELFR	7
Q96KN2	Beta-Ala-His dipeptidase	ALEQDLPVNIK	11

## Discussion

In this comparison of serum from matched controls and subjects in whom the early stages in the development of carotid plaques were detected, the proteomic analysis indicated the differential abundance of several proteins. Amongst these were a number of proteins that have been previously linked with atherosclerosis, i.e. APOC3, APOE, CNDP1, CDH13, GSN, MMP2 and fibulin1^8^. Using a machine learning approach the combination of FBLN1C, APOE and CDH13 was found to provide the best classification of the cases from controls. A targeted SRM assay was subsequently developed for the measurement of these differentially abundant proteins. Due to the unavailability of a similar sample set, it was used for verification only in the same samples as analyzed in the discovery phase. The verification measurements were performed for the samples prepared without depletion. The use of undepleted samples removed potential biases created by the depletion step and succeeded against the background of abundant serum proteins due to the intrinsic sensitivity of the targeted method. Based on this verification data, the quantitative difference of FBLN1C remained significant. The failure to confirm the dissimilarities detected for APOE and CDH13 in the discovery data could reflect the small magnitude and variability of these intra-individual differences.

The Fibulins are a family of six moderately abundant serum proteins (FBLN1 – FBLN6) that are linked with the extracellular matrix (ECM) proteins^18^. Differences in FBLN1 abundance have previously been observed in several studies, including its relationship to atherosclerosis, cardiovascular risk, arterial stiffness and type 2 diabetes (T2D). For example, Kawata *et al*. first reported reduced plasma levels of FLBN1 in patients with acute myocardial infarction and stable angina^19^. Both lower and higher levels have been reported in the plasma of T2D patients^20,21^, but differences in the duration of the disease (recently diagnosed vs. established disease) could reflect upon the latter division. In relationship to cardiovascular disease, FLBN1 was detected as a component of atherosclerotic lesions, and Argraves *et al*. suggested that decreased plasma FBLN1 could reflect its accumulation in the plaque^22^. Similarly, the accumulation of FBLN1 in the arterial wall has been detected in patients with T2D^21^, although in a situation in which the FBLN1 plasma levels were higher than in the matched controls. In contrast, in newly diagnosed T2D patients, lower plasma FBLN1 was found and correlated with carotid-femoral arterial stiffness^20^. On the basis of the latter observation, Paapstel *et al*. studied the relationship between arterial stiffness and serum FBLN1 levels in patients with atherosclerosis^23^. Here they found higher levels of FBLN1 in the patients.

In addition to the complex interplay between arterial stiffness and atherosclerotic risk factors^24^, the relationship between plasma levels of FBLN1 and the early stages of plaque formation is contradictory and yet to be clearly established.

In the above examples, there are differences in the study sizes, diseases and duration, as well as specificity of the controls. Further, whilst alternative splicing produces four FBLN1 proteoforms (A, B, C and D)^25^, the former the studies have not made any distinction between these. These variants may differ from both a structural and functional perspective. In this respect their distinction as proteoforms and this terminology implicates protein variants that are not coded explicitly in the genome, i.e. including alternative RNA splicing and post-translational modifications^26^. Our proteomic data has specifically highlighted significant differences for the C proteoform. Fibulin1C has been reported to be the predominant form in plasma^25^ and has been identified in the tissue secretome analysis of coronary arteries^27^. Within the limitations of this knowledge, we can only speculate whether this difference in abundance represents a phenotype that is more susceptible to plaque formation or an early indication of onset. Potentially, it may be that the structural differences for this specific proteoform of FBLN1 could, following some trigger, contribute to its interaction with extracellular matrix protein (ECM) molecules and accumulation on the arterial intimal walls, and thus be reflected by its lower serum abundance.

The progression of the atherosclerotic process is influenced by a range of factors including age, diet, stress and other risk factors such as smoking^28,29^. In the present study the subjects were carefully matched by age, gender, BMI and blood pressure. However, seven cases and five controls were smokers, and the influence of smoking was not separately evaluated. Additional limitations of the current study is the sample size and the need for further validation studies in a larger independent cohort. Furthermore, structural studies explaining the interaction of the FBLN1C proteoform with ECM proteins could provide insights into its potential role in plaque formation.

In summary, from these measurements from a cohort selected to study the risk and development of atherosclerotic lesions, distinguishing proteomics profiles were identified in subjects showing early signs of plaque development. In particular, FBLN1C is implicated as a target for further investigation

## Methods

### Study Population and design

The samples were selected from participants in the Cardiovascular Risk in Young Finns cohort^15^. On the basis of carotid ultrasound measurements, samples from 43 individuals with a distinct carotid artery plaque were selected together with samples from 43 controls. The controls were matched by age, sex, body weight and systolic blood pressure. The age range of the subjects was 30–45 years. The study design is depicted in Fig. 1, and the clinical characteristics of cases and controls are shown in Table 1. The measurements and data included in this manuscript have been acquired following the guidelines of the Declaration of Helsinki for research on human participants and were conducted with the permission of the Ethical Committees of the University Hospitals of Turku with written informed consent.

### Carotid intima-media thickness measurement

Ultrasound examination of the left carotid artery, including common carotid artery and carotid bifurcation, were performed using B-mode ultrasound (Acuson Sequoia 512, Siemens) with the 13.0-MHz linear-array transducer, according to a standardized protocol^30^. Intima-media thickness was measured from digitally stored scans by one reader blinded to participant details. The best-quality end-diastolic frame was selected, and ultrasonic calipers were used to measure carotid intima-media thickness from the far wall of the common carotid artery 10 mm proximal to the bifurcation. To detect the carotid plaques, the images were scanned and the presence of atherosclerotic plaque defined as a distinct area of the carotid vessel wall protruding into the lumen >50% of the adjacent intima-media layer^31^. All the observed plaques were detected in the carotid bifurcation.

### ApoE genotype determination

*APOE* genotyping was performed by using TaqMan SNP Genotyping Assays (rs429358 assay C 3084793_20; rs7412 assay C_904973_10) and the ABI Prism 7900HT Sequence Detection System (Applied Biosystems, Foster City, CA, USA).

### Sample preparation

#### Immunodepletion of high abundant proteins

An Agilent MARS-14 immunoaffinity column was used for the targeted removal of the most abundant serum proteins. The isolated, lower abundance proteins were reduced, alkylated, digested and desalted prior to mass spectrometry (MS) analysis as described previously^32^.

#### Preparation of undepleted serum

For the verification measurements, the serum samples were diluted in the denaturant, reduced, alkylated and digested using sequencing grade modified trypsin (Promega)^32^.

Heavy labeled synthetic analogs of proteotypic peptides^33^ of the differentially abundant proteins, housekeeping proteins (Alpha-1B-glycoprotein and complement C1s subcomponent) and the known CVD risk factor, Apolipoprotein B-100, were spiked into the digests together with indexed retention time standard (iRT) peptides (Biognosys). These were selected from discovery phase peptide data with the consideration of the consistent detection, absence of potentially modified residues and missed cleavages (Table 3). The heavy-labeled synthetic analogs (lysine ^13^C_6_^15^N_2_ and arginine ^13^C_6_^15^N_4_) equivalent of proteotypic peptides were obtained (Thermo Fischer Scientific).

### Mass spectrometry analysis

#### Discovery phase

Aliquots of the depleted serum digests (500 ng) were analyzed with an Easy-nLC-II coupled to a LTQ Orbitrap Velos Pro mass spectrometer (Thermo Fisher Scientific). The peptides were separated on 150 mm × 75 µm ID column packed with 5 µm magic C18-bonded silica (200 Å). The peptides were eluted with an increasing gradient of 5–35% acetonitrile at a flow rate of 300 nl/min using a binary mixture of water and acetonitrile with 0.2% formic acid. The mass spectrometer was operated in data-dependent acquisition mode with a selection of top 15 precursors followed by fragmentation using collisional induced dissociation (CID) method. All the samples were analyzed in quadruplicate as randomized batches^32^. To ensure comparable instrument performance throughout the time span of the discovery phase study, an in-house standard was periodically analyzed to establish the consistency of the signal intensity and chromatographic separation (Supplementary Figure S1).

#### Verification

Aliquots of the digested peptides (250 ng), spiked with heavy labeled peptides and index retention time (iRT) peptides, were analyzed with Easy-nLC-II coupled to a TSQ Vantage mass spectrometer (Thermo Fisher Scientific)^32^. The peptide mixture was separated with a 150 mm × 75 µm ID column packed with ReproSil-Pur C18-AQ 5 µm resin (Dr. Maisch GmbH). An unscheduled analysis of the sample was carried out to generate iRT values of target peptides and their heavy counterparts. Skyline software was used to build up the scheduled method for the selected targets using the unscheduled run. The scheduled method was then edited by removing interfering signals^32^ to monitor 99 transitions from 33 peptides, representing 10 proteins and the iRT peptides.

The 86 serum samples (N = 43 vs. 43) were prepared without depletion and analyzed as randomized batches. To monitor the variation of peak areas and retention time across and within the batches, a pooled digest of the undepleted serum was included in each batch.

### Data processing

#### Protein informatics analysis (Discovery phase)

The tandem mass spectra data were searched against a UniProt human isoform protein sequence database (UniProt release, August 2017, entries = 42,210) using the Andromeda^34^ search algorithm and MaxQuant 1.5.5.1^35^. The search parameters were set to allow two missed tryptic cleavages, methyl methanethiosulfonate (MMTS) modification of cysteine and variable modification of methionine and acetylation of the protein N terminus. A false discovery rate (FDR) of 1% was applied at peptide and protein level. The “match between run” option (matching and alignment time window = 0.7 and 20 minutes respectively) was selected in order to enable the transfer of identifications across the mass spectrometric measurements^36^. The label-free normalized intensity values (MaxQuant output) were further analyzed using Perseus software^36,37^. Briefly, the output was filtered to exclude reverse hits and proteins only inferred by the detection of single variable modifications. Furthermore, only proteins identified with >1 unique plus razor peptides were considered. The razor peptides are defined as those that are shared between different protein groups and are assigned to the protein that has the most peptides^35,38^. The data was then log_2_ transformed followed by filtering to at least 50% valid values. Missing values were imputed by “imputation from normal distribution” (width = 0.3, downshift = 1.8)^39^ followed by taking the average of quadruplicate analyses. The subsequent statistical analysis of data was then performed using R^40^.

#### SRM data analysis and transition selection

Skyline version 4.1 was used to develop and analyze SRM assay transitions^41^. The quality of the transitions and confirmation of light/heavy pairs were manually inspected. On account of interferences in the transitions for one of the FBLN1C peptides, DLLLTVK alone was considered for its statistical analysis in the SRM data. Out of the two housekeeping proteins included for normalization of the data, the TNFDNDIALVR peptide from complement C1s subcomponent was used.

### Statistical analysis

#### Reproducibility-optimized test statistic (ROTS)

The label-free normalized protein intensity abundance values obtained from MaxQuant analysis were used as input for ROTS analysis^36,42,43^. Briefly, the log2 transformed data were analyzed using non-parametric method relying on the family of t-type statistics which ranks the proteins based on their differential expression in two group conditions and the calculation was made with 1000 permutations (FDR < 0.05).

#### Machine learning classification

To identify the protein panel with the highest discriminative performance, Lasso penalized logistic regression^44^, implemented in the R package *glmnet*^45^, was applied to the serum proteomics data. First, all candidate predictors were identified by shrinking the coefficients of non-informative predictors to zero using Lasso with 3-fold cross-validation, repeating the randomization procedure 200 times. In each fold, only significantly differentially abundant proteins (ROTS; P < 0.05) were considered. Finally, among the top 20 most frequent candidate proteins, the Lasso model with the protein panel having the largest improvement in discriminative performance in terms of area under the receiver-operating characteristic curve (AUROC) and with the least number of predictors was identified. Statistical significance of the differences in the AUROC values between the models was determined using the DeLong method^46^ implemented in the R package pROC^47^.

#### MSStats

The MSStats (3.8.4) plugin included in the Skyline software was used for the group comparison between cases and controls^48^. Briefly, after normalizing the data to the housekeeping protein the statistics were calculated on the basis of the Turkey’s median polish method. The latter uses a linear mixed model to give a robust estimation of differentially abundant proteins between conditions.

### Data availability

The LC-MS/MS proteomics discovery data are available from the ProteomeXchange Consortium via the PRIDE^49^ partner repository with the dataset identifier PXD008278. The SRM verification data are available from the ProteomeXchange Consortium via the PASSEL^50^ partner repository with dataset identifier PASS01146.

## Electronic supplementary material

Supplementry file
