# Supplementary material for: Serum Proteomic Profiling to Identify Biomarkers of Premature Carotid Atherosclerosis
Source: Sci Rep. 2018 Jun 15;8:9209. doi: 10.1038/s41598-018-27265-9 (PMC6003912; doi:10.1038/s41598-018-27265-9)
Supplement: Supplementary file 1 — Supplementry file [file 41598_2018_27265_MOESM1_ESM.docx]

**Serum Proteomic Profiling to Identify Biomarkers of Premature Carotid Atherosclerosis**

Santosh D. Bhosale^1^, Robert Moulder^1^, Mikko S. Venäläinen^1^, Juhani S. Koskinen ^2,6^, Niina Pitkänen ^6^, Markus T Juonala^2^, Mika A.P. Kähönen^3^, Terho J. Lehtimäki^4^, Jorma S.A. Viikari^2^, Laura L. Elo^1^, David R. Goodlett^1,5^, Riitta Lahesmaa^1^*, Olli T. Raitakari^6,7^*

**SUPPLEMENTARY DATA:**

**Supplementary Figure S1.** Line chart showing the performance of in-house standard (Carbonic anhydrase) to maintain instrument performance.

**
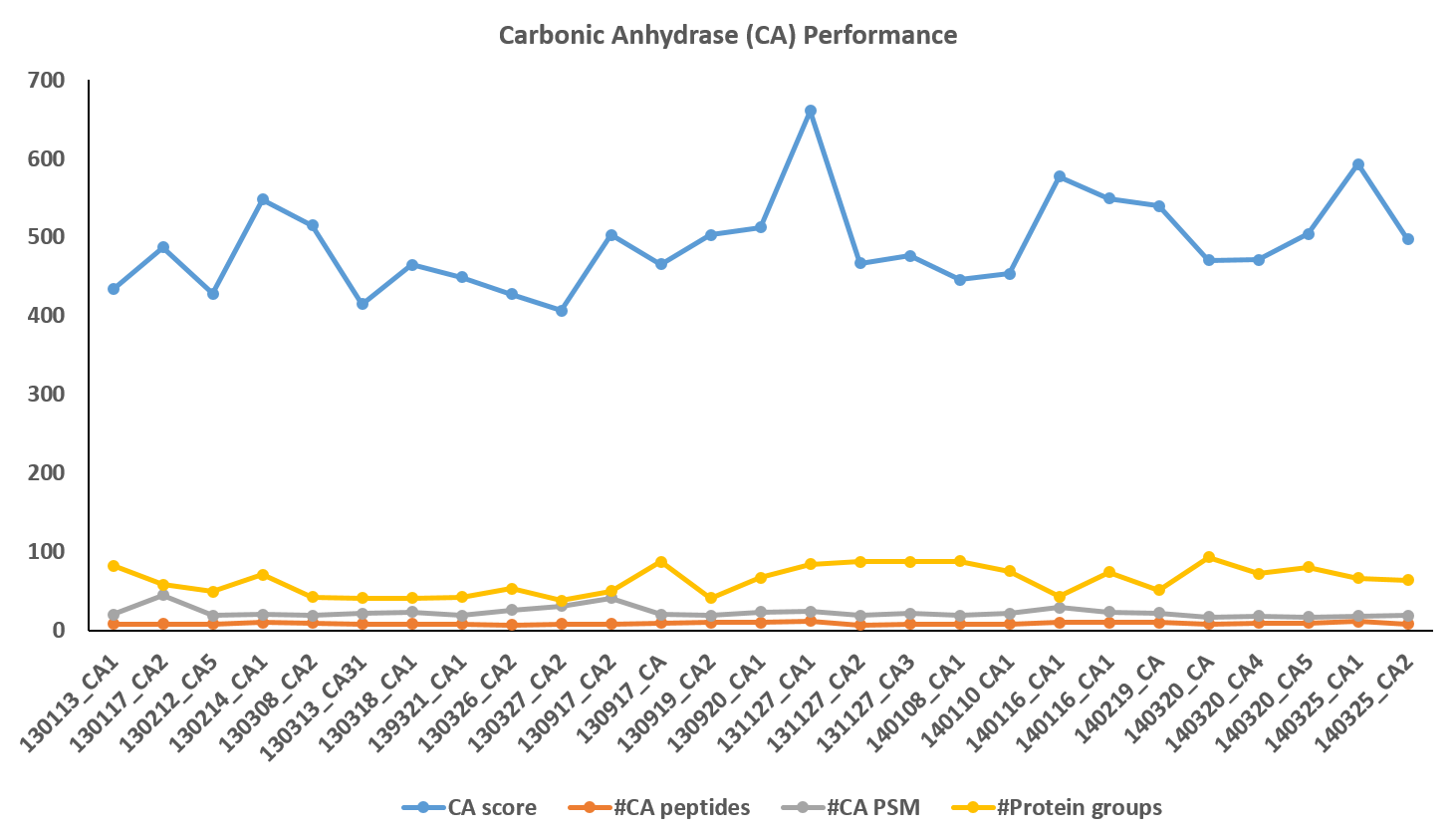
**

**Supplementary Figure S2.** A) Frequency of distribution of apolipoprotein E (ApoE) alleles. p value is >0.05, thus no statistical difference between the cases and controls. B) The distribution of apoE risk genotype for carotid atherosclerosis in the cases and controls. p value is >0.05, thus no statistical difference between carriers and non-carriers. The p-value is calculated by Fisher's Exact Test for Count Data.

**A)**

**
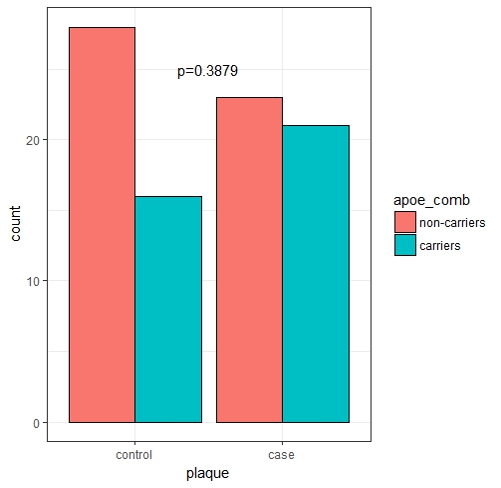
**

**B)**

**
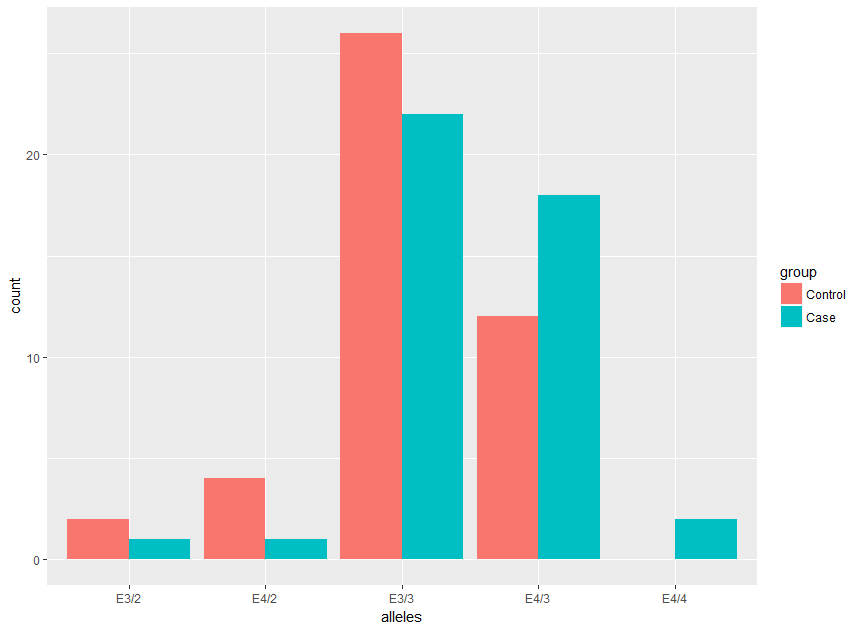
**

p=0.229

**Supplementary Figure S3.**

**Receiver operating characteristics (ROC) curve of the SRM-MS data for the panel.** P23142-4 (FBLN1C) alone classified cases from controls with AUROC = 0.64 (95% CI: 0.53-0.76)**.** The addition of P02649 (APOE) and P55290 (CDH13) to FBLN1C improved AUROC to 0.66 (95% CI: 0.54-0.78) (p = 0.8). (N = 43 vs. 43).

**
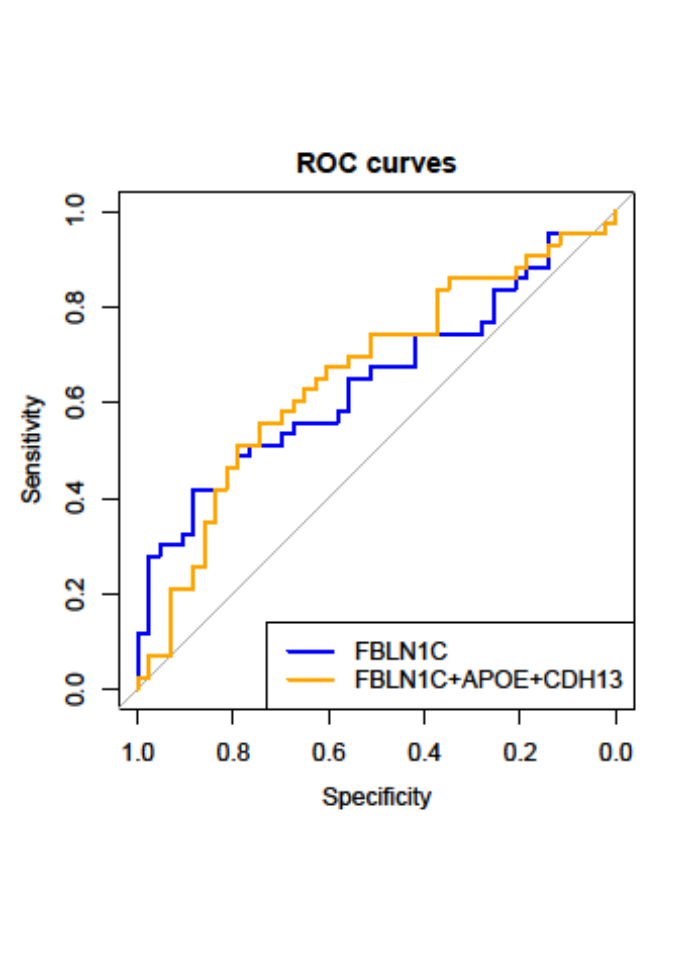
**

**Supplementary table.**

**Supplementary Table1.**

Proteins identified with >1 razors + unique peptides.

| **Majority protein IDs** | **Protein names** | **Razor + unique peptides** | **Sequence coverage [%]** |
| --- | --- | --- | --- |
| P04114 | Apolipoprotein B-100 | 404 | 72.8 |
| P0C0L5 | Complement C4-B | 169 | 77.7 |
| P01024 | Complement C3 | 157 | 81.9 |
| P02751 | Fibronectin | 128 | 62.9 |
| P08603 | Complement factor H | 111 | 73.7 |
| P00747 | Plasminogen | 103 | 84.8 |
| P00450 | Ceruloplasmin | 97 | 82.1 |
| P01023 | Alpha-2-macroglobulin | 94 | 63.1 |
| P01031 | Complement C5 | 91 | 58.8 |
| P00751 | Complement factor B | 83 | 68.6 |
| Q14624 | Inter-alpha-trypsin inhibitor heavy chain H4 | 82 | 64.8 |
| P06727 | Apolipoprotein A-IV | 80 | 93.9 |
| P02774 | Vitamin D-binding protein | 76 | 93.7 |
| P06396 | Gelsolin | 70 | 75.8 |
| P43652 | Afamin | 68 | 72.6 |
| P02768 | Serum albumin | 67 | 90.6 |
| P01042 | Kininogen-1 | 58 | 50.6 |
| P02787 | Serotransferrin | 58 | 69.2 |
| P13671 | Complement component C6 | 58 | 51.1 |
| P19823 | Inter-alpha-trypsin inhibitor heavy chain H2 | 58 | 50.7 |
| P00734 | Prothrombin | 57 | 65.6 |
| P02790 | Hemopexin | 55 | 78.8 |
| P10643 | Complement component C7 | 54 | 57.5 |
| P01008 | Antithrombin-III | 51 | 75.4 |
| P07996 | Thrombospondin-1 | 51 | 41.6 |
| P19827 | Inter-alpha-trypsin inhibitor heavy chain H1 | 51 | 53.7 |
| P12259 | Coagulation factor V | 48 | 25.2 |
| P00738 | Haptoglobin | 46 | 78.8 |
| P03952 | Plasma kallikrein | 46 | 68.7 |
| P00736 | Complement C1r subcomponent | 45 | 58.3 |
| P04264 | Keratin, type II cytoskeletal 1 | 44 | 62.7 |
| P09871 | Complement C1s subcomponent | 44 | 60.8 |
| P04275 | von Willebrand factor | 43 | 18.6 |
| Q06033 | Inter-alpha-trypsin inhibitor heavy chain H3 | 42 | 49.5 |
| P04196 | Histidine-rich glycoprotein | 41 | 59.6 |
| P01009 | Alpha-1-antitrypsin | 38 | 62.2 |
| P05156 | Complement factor I | 38 | 50.6 |
| P08519 | Apolipoprotein(a) | 38 | 49.7 |
| P10909 | Clusterin | 38 | 56.2 |
| P01011 | Alpha-1-antichymotrypsin | 37 | 65 |
| P04217 | Alpha-1B-glycoprotein | 37 | 69.3 |
| P05155 | Plasma protease C1 inhibitor | 37 | 54.2 |
| P13645 | Keratin, type I cytoskeletal 10 | 36 | 61.4 |
| P00748 | Coagulation factor XII | 36 | 48.1 |
| P06681 | Complement C2 | 36 | 50.1 |
| P25311 | Zinc-alpha-2-glycoprotein | 35 | 71.1 |
| P02649 | Apolipoprotein E | 34 | 80.1 |
| P05546 | Heparin cofactor 2 | 34 | 60.9 |
| P35858 | Insulin-like growth factor-binding protein complex acid labile subunit | 34 | 51.7 |
| P36955 | Pigment epithelium-derived factor | 34 | 72 |
| P02647 | Apolipoprotein A-I | 33 | 77.2 |
| P02749 | Beta-2-glycoprotein 1 | 33 | 69.9 |
| P07358 | Complement component C8 beta chain | 33 | 59.1 |
| P35527 | Keratin, type I cytoskeletal 9 | 33 | 58.4 |
| P02760 | Protein AMBP | 32 | 64.2 |
| P02748 | Complement component C9 | 30 | 48.1 |
| P35908 | Keratin, type II cytoskeletal 2 epidermal | 30 | 67.1 |
| P04004 | Vitronectin | 29 | 47.7 |
| P05160 | Coagulation factor XIII B chain | 29 | 46.3 |
| Q96KN2 | Beta-Ala-His dipeptidase | 29 | 57.6 |
| P03951 | Coagulation factor XI | 28 | 57 |
| P02765 | Alpha-2-HS-glycoprotein | 27 | 58.9 |
| P07357 | Complement component C8 alpha chain | 27 | 55.5 |
| P08697 | Alpha-2-antiplasmin | 27 | 57.4 |
| P26927 | Hepatocyte growth factor-like protein | 27 | 51.2 |
| Q92954 | Proteoglycan 4 | 27 | 33 |
| Q9UK55 | Protein Z-dependent protease inhibitor | 27 | 58.3 |
| P29622 | Kallistatin | 26 | 59.5 |
| Q96PD5 | N-acetylmuramoyl-L-alanine amidase | 26 | 62.7 |
| P22105 | Tenascin-X | 25 | 9.6 |
| P23142 | Fibulin-1 | 25 | 37.1 |
| Q16610 | Extracellular matrix protein 1 | 25 | 53.5 |
| P51884 | Lumican | 24 | 49.4 |
| O00533 | Neural cell adhesion molecule L1-like protein | 23 | 21.9 |
| P02671 | Fibrinogen alpha chain | 23 | 24.6 |
| P07225 | Vitamin K-dependent protein S | 23 | 34.6 |
| P04003 | C4b-binding protein alpha chain | 22 | 37.7 |
| P01019 | Angiotensinogen | 21 | 55.1 |
| P02753 | Retinol-binding protein 4 | 21 | 83.6 |
| P80108 | Phosphatidylinositol-glycan-specific phospholipase D | 21 | 31.3 |
| Q04756 | Hepatocyte growth factor activator | 21 | 35.7 |
| P05543 | Thyroxine-binding globulin | 20 | 51.3 |
| P13796 | Plastin-2 | 20 | 40.7 |
| Q14520 | Hyaluronan-binding protein 2 | 20 | 40.3 |
| P01871 | Ig mu chain C region | 19 | 46.8 |
| P04278 | Sex hormone-binding globulin | 19 | 58 |
| P06276 | Cholinesterase | 18 | 35 |
| P08185 | Corticosteroid-binding globulin | 18 | 52.1 |
| P48740 | Mannan-binding lectin serine protease 1 | 18 | 35.3 |
| O75882 | Attractin | 17 | 15.6 |
| P02750 | Leucine-rich alpha-2-glycoprotein | 17 | 50.1 |
| P22792 | Carboxypeptidase N subunit 2 | 17 | 45.7 |
| Q8WZ42 | Titin | 17 | 1 |
| P02775 | Platelet basic protein | 16 | 60.9 |
| P05452 | Tetranectin | 16 | 55.4 |
| P09172 | Dopamine beta-hydroxylase | 16 | 36.5 |
| P15169 | Carboxypeptidase N catalytic chain | 16 | 48 |
| P98160 | Basement membrane-specific heparan sulfate proteoglycan core protein | 16 | 5.6 |
| Q96IY4 | Carboxypeptidase B2 | 16 | 46.3 |
| P04070 | Vitamin K-dependent protein C | 15 | 37.7 |
| P27918 | Properdin | 15 | 32 |
| P33151 | Cadherin-5 | 15 | 24 |
| Q9UGM5 | Fetuin-B | 15 | 44.2 |
| Q9Y6R7 | IgGFc-binding protein | 15 | 6.2 |
| O75636 | Ficolin-3 | 14 | 54.5 |
| P00742 | Coagulation factor X | 14 | 29.7 |
| P01876 | Ig alpha-1 chain C region | 14 | 57.8 |
| P05154 | Plasma serine protease inhibitor | 14 | 36.7 |
| P11021 | 78 kDa glucose-regulated protein | 14 | 24.3 |
| P17936 | Insulin-like growth factor-binding protein 3 | 14 | 45 |
| P49747 | Cartilage oligomeric matrix protein | 14 | 30.8 |
| P68871 | Hemoglobin subunit beta | 14 | 89.8 |
| O00391 | Sulfhydryl oxidase 1 | 13 | 32.8 |
| P08571 | Monocyte differentiation antigen CD14 | 13 | 45.6 |
| Q9BXR6 | Complement factor H-related protein 5 | 13 | 29.9 |
| O43866 | CD5 antigen-like | 12 | 46.7 |
| P01860 | Ig gamma-3 chain C region | 12 | 44.3 |
| P02652 | Apolipoprotein A-II | 12 | 77 |
| P02743 | Serum amyloid P-component | 12 | 48.9 |
| P05062 | Fructose-bisphosphate aldolase B | 12 | 44.8 |
| P27169 | Serum paraoxonase/arylesterase 1 | 12 | 51.3 |
| P43251 | Biotinidase | 12 | 28.5 |
| P49908 | Selenoprotein P | 12 | 24.1 |
| P63261 | Actin, cytoplasmic 2 | 12 | 38.1 |
| Q9NQ79 | Cartilage acidic protein 1 | 12 | 29 |
| P00746 | Complement factor D | 11 | 60.5 |
| P02746 | Complement C1q subcomponent subunit B | 11 | 49.4 |
| P02766 | Transthyretin | 11 | 69.4 |
| P07360 | Complement component C8 gamma chain | 11 | 55 |
| P22352 | Glutathione peroxidase 3 | 11 | 39.8 |
| P40197 | Platelet glycoprotein V | 11 | 28.4 |
| Q9NZP8 | Complement C1r subcomponent-like protein | 11 | 27.3 |
| P00488 | Coagulation factor XIII A chain | 10 | 16.4 |
| P02747 | Complement C1q subcomponent subunit C | 10 | 38 |
| P06702 | Protein S100-A9 | 10 | 85.1 |
| P43121 | Cell surface glycoprotein MUC18 | 10 | 17.8 |
| P55056 | Apolipoprotein C-IV | 10 | 52.8 |
| P69905 | Hemoglobin subunit alpha | 10 | 85.9 |
| Q03591 | Complement factor H-related protein 1 | 10 | 71.2 |
| Q08380 | Galectin-3-binding protein | 10 | 22.1 |
| Q15582 | Transforming growth factor-beta-induced protein ig-h3 | 10 | 18.2 |
| Q6UXB8 | Peptidase inhibitor 16 | 10 | 28.3 |
| O14791 | Apolipoprotein L1 | 9 | 26.6 |
| O95445 | Apolipoprotein M | 9 | 60.3 |
| P00740 | Coagulation factor IX | 9 | 22.5 |
| P01834 | Ig kappa chain C region | 9 | 86.9 |
| P05067 | Amyloid beta A4 protein | 9 | 14.6 |
| P09486 | SPARC | 9 | 37.3 |
| P11226 | Mannose-binding protein C | 9 | 41.5 |
| P15144 | Aminopeptidase N | 9 | 12 |
| P18428 | Lipopolysaccharide-binding protein | 9 | 18.1 |
| P19320 | Vascular cell adhesion protein 1 | 9 | 17.9 |
| P35542 | Serum amyloid A-4 protein | 9 | 55.4 |
| Q12860 | Contactin-1 | 9 | 11.6 |
| Q13822 | Ectonucleotide pyrophosphatase/phosphodiesterase family member 2 | 9 | 10.5 |
| Q15485 | Ficolin-2 | 9 | 28.8 |
| P02656 | Apolipoprotein C-III | 8 | 62.6 |
| P02776 | Platelet factor 4 | 8 | 44.6 |
| P02788 | Lactotransferrin | 8 | 15.8 |
| P07359 | Platelet glycoprotein Ib alpha chain | 8 | 11.8 |
| P08637 | Low affinity immunoglobulin gamma Fc region receptor III-A | 8 | 27.2 |
| P08709 | Coagulation factor VII | 8 | 25.2 |
| P0DOY3 | Ig lambda-6 chain C region | 8 | 74.5 |
| P14151 | L-selectin | 8 | 21.2 |
| P22891 | Vitamin K-dependent protein Z | 8 | 19.8 |
| P32119 | Peroxiredoxin-2 | 8 | 38.9 |
| Q13201 | Multimerin-1 | 8 | 10.7 |
| Q14515 | SPARC-like protein 1 | 8 | 15.8 |
| Q6EMK4 | Vasorin | 8 | 17.8 |
| P00915 | Carbonic anhydrase 1 | 7 | 43.7 |
| P01042 | Kininogen-1 | 7 | 62.3 |
| P02654 | Apolipoprotein C-I | 7 | 39.8 |
| P02655 | Apolipoprotein C-II | 7 | 59.4 |
| P0C0L4 | Complement C4-A | 7 | 77.8 |
| P13591 | Neural cell adhesion molecule 1 | 7 | 14.6 |
| P36980 | Complement factor H-related protein 2 | 7 | 65 |
| P48740 | Mannan-binding lectin serine protease 1 | 7 | 39.8 |
| P61626 | Lysozyme C | 7 | 53.4 |
| Q02985 | Complement factor H-related protein 3 | 7 | 31.6 |
| Q15113 | Procollagen C-endopeptidase enhancer 1 | 7 | 24.9 |
| Q76LX8 | A disintegrin and metalloproteinase with thrombospondin motifs 13 | 7 | 7.3 |
| Q7Z7G0 | Target of Nesh-SH3 | 7 | 18.7 |
| O00187 | Mannan-binding lectin serine protease 2 | 6 | 37.8 |
| O43493 | Trans-Golgi network integral membrane protein 2 | 6 | 17.5 |
| P02745 | Complement C1q subcomponent subunit A | 6 | 29.4 |
| P04180 | Phosphatidylcholine-sterol acyltransferase | 6 | 22.7 |
| P08294 | Extracellular superoxide dismutase [Cu-Zn] | 6 | 47.5 |
| P18065 | Insulin-like growth factor-binding protein 2 | 6 | 27.1 |
| P22692 | Insulin-like growth factor-binding protein 4 | 6 | 25.2 |
| P23142-4 | Fibulin 1 proteoform C | 6 | 36.6 |
| P23470 | Receptor-type tyrosine-protein phosphatase gamma | 6 | 7.9 |
| P41222 | Prostaglandin-H2 D-isomerase | 6 | 40 |
| P61769 | Beta-2-microglobulin | 6 | 63.9 |
| P62328 | Thymosin beta-4 | 6 | 90.9 |
| P80723 | Brain acid soluble protein 1 | 6 | 50.7 |
| Q6UWP8 | Suprabasin | 6 | 25.3 |
| Q9BWP8 | Collectin-11 | 6 | 31 |
| Q9UNW1 | Multiple inositol polyphosphate phosphatase 1 | 6 | 15.4 |
| Q86YZ3 | Hornerin | 5 | 10.5 |
| O95497 | Pantetheinase | 5 | 16.6 |
| P03950 | Angiogenin | 5 | 23.8 |
| P04075 | Fructose-bisphosphate aldolase A | 5 | 17 |
| P05109 | Protein S100-A8;Protein S100-A8, N-terminally processed | 5 | 45.2 |
| P05362 | Intercellular adhesion molecule 1 | 5 | 9.8 |
| P14543 | Nidogen-1 | 5 | 7.6 |
| P15151 | Poliovirus receptor | 5 | 14.8 |
| P24593 | Insulin-like growth factor-binding protein 5 | 5 | 15.1 |
| P54108 | Cysteine-rich secretory protein 3 | 5 | 21.2 |
| P55290 | Cadherin-13 | 5 | 9.3 |
| Q6UX71 | Plexin domain-containing protein 2 | 5 | 11.3 |
| Q9NPH3 | Interleukin-1 receptor accessory protein | 5 | 16.5 |
| P13647 | Keratin, type II cytoskeletal 5 | 4 | 16.8 |
| P00918 | Carbonic anhydrase 2 | 4 | 25.4 |
| P01034 | Cystatin-C | 4 | 31.5 |
| P02741 | C-reactive protein;C-reactive protein(1-205) | 4 | 38.5 |
| P02763 | Alpha-1-acid glycoprotein 1 | 4 | 23.9 |
| P07195 | L-lactate dehydrogenase B chain | 4 | 14.1 |
| P12955 | Xaa-Pro dipeptidase | 4 | 14.4 |
| P16070 | CD44 antigen | 4 | 13.3 |
| P20851 | C4b-binding protein beta chain | 4 | 20.7 |
| P24592 | Insulin-like growth factor-binding protein 6 | 4 | 27.9 |
| P55058 | Phospholipid transfer protein | 4 | 14.1 |
| Q01459 | Di-N-acetylchitobiase | 4 | 17.7 |
| Q12913 | Receptor-type tyrosine-protein phosphatase eta | 4 | 12.1 |
| Q13093 | Platelet-activating factor acetylhydrolase | 4 | 10.2 |
| Q14624 | Inter-alpha-trypsin inhibitor heavy chain H4 | 4 | 67 |
| Q7Z7M0 | Multiple epidermal growth factor-like domains protein 8 | 4 | 3 |
| Q86U17 | Serpin A11 | 4 | 11.6 |
| Q86UD1 | Out at first protein homolog | 4 | 16.8 |
| Q99969 | Retinoic acid receptor responder protein 2 | 4 | 31.9 |
| Q9Y5Y7 | Lymphatic vessel endothelial hyaluronic acid receptor 1 | 4 | 8.1 |
| O75015 | Low affinity immunoglobulin gamma Fc region receptor III-B | 3 | 29.2 |
| P01033 | Metalloproteinase inhibitor 1 | 3 | 22.7 |
| P01344 | Insulin-like growth factor II | 3 | 19.4 |
| P01591 | Immunoglobulin J chain | 3 | 23.3 |
| P05019 | Insulin-like growth factor I | 3 | 30.7 |
| P08253 | 72 kDa type IV collagenase | 3 | 7.4 |
| P10124 | Serglycin | 3 | 18.4 |
| P13473 | Lysosome-associated membrane glycoprotein 2 | 3 | 7.1 |
| P13598 | Intercellular adhesion molecule 2 | 3 | 14.9 |
| Q10588 | ADP-ribosyl cyclase/cyclic ADP-ribose hydrolase 2 | 3 | 12.7 |
| Q12805 | EGF-containing fibulin-like extracellular matrix protein 1 | 3 | 8.5 |
| Q16627 | C-C motif chemokine 14 | 3 | 43 |
| Q7Z5P9 | Mucin-19 | 3 | 0.8 |
| Q8NBP7 | Proprotein convertase subtilisin/kexin type 9 | 3 | 7.7 |
| P17813 | Endoglin | 2 | 4.5 |
| P59666 | Neutrophil defensin 3 | 2 | 19.1 |
| Q13103 | Secreted phosphoprotein 24 | 2 | 10.4 |
| Q15848 | Adiponectin | 2 | 12.3 |

**Supplementary Table2. Frequency of distribution of apolipoprotein E (ApoE) alleles.**

| **ApoE alleles** | **Control** | **Case** |
| --- | --- | --- |
| E3/2 | 2 | 1 |
| E4/2 | 4 | 1 |
| E3/3 | 26 | 22 |
| E4/3 | 12 | 18 |
| E4/4 | 0 | 2 |
